# Adaptive radiotherapy for head and neck cancer reduces the requirement for rescans during treatment due to spinal cord dose

**DOI:** 10.1186/s13014-019-1400-3

**Published:** 2019-11-01

**Authors:** Louise Belshaw, Christina E. Agnew, Denise M. Irvine, Keith P. Rooney, Conor K. McGarry

**Affiliations:** 1 Radiotherapy Physics, Northern Ireland Cancer Centre, Belfast City Hospital, Belfast, Northern Ireland; 2 Clinical Oncology, Northern Ireland Cancer Centre, Belfast City Hospital, Belfast, Northern Ireland; 3 Centre for Cancer Research and Cell Biology, Queen’s University Belfast, Belfast, Northern Ireland

**Keywords:** Adaptive radiotherapy, Radiotherapy, Head and neck, Deformable image registration, Spinal cord, Radiation dose, Image guided radiotherapy

## Abstract

**Background:**

Patients treated with radiotherapy for head and neck (H&N) cancer often experience anatomical changes. The potential compromises to Planning Target Volume (PTV) coverage or Organ at Risk (OAR) sparing has prompted the use of adaptive radiotherapy (ART) for these patients. However, implementation of ART is time and resource intensive. This study seeks to define a clinical trigger for H&N re-plans based on spinal cord safety using kV Cone-Beam Computed Tomography (CBCT) verification imaging, in order to best balance clinical benefit with additional workload.

**Methods:**

Thirty-one H&N patients treated with Volumetric Modulated Arc Therapy (VMAT) who had a rescan CT (rCT) during treatment were included in this study. Contour volume changes between the planning CT (pCT) and rCT were determined. The original treatment plan was calculated on the pCT, CBCT prior to the rCT, pCT deformed to the anatomy of the CBCT (dCT), and rCT (considered the gold standard). The dose to 0.1 cc (D0.1cc) spinal cord was evaluated from the Dose Volume Histograms (DVHs).

**Results:**

The median dose increase to D0.1cc between the pCT and rCT was 0.7 Gy (inter-quartile range 0.2–1.9 Gy, *p* < 0.05). No correlation was found between contour volume changes and the spinal cord dose increase. Three patients exhibited an increase of 7.0–7.2 Gy to D0.1cc, resulting in a re-plan; these patients were correctly identified using calculations on the CBCT/dCT.

**Conclusions:**

An adaptive re-plan can be triggered using spinal cord doses calculated on the CBCT/dCT. Implementing this trigger can reduce patient appointments and radiation dose by eliminating up to 90% of additional un-necessary CT scans, reducing the workload for radiographers, physicists, dosimetrists, and clinicians.

## Background

Intensity Modulated Radiation Therapy (IMRT) and Volumetric Modulated Arc Therapy (VMAT) have become the standard of care for patients receiving radiotherapy for cancer of the head and neck, due to the possibility of sculpting the dose distribution around the PTV and sparing the surrounding healthy tissues and organs at risk (OARs) [1–3]. Reproducible and accurate positioning of the patient on each treatment fraction is essential for accurate treatment delivery, as even small changes from the reference anatomy of the patient’s planning CT scan (pCT) have the potential to compromise the PTV coverage or sparing of healthy tissues. The use of patient immobilisation devices, such as thermoplastic shells, mouth bites, and handgrips, along with image-guided radiotherapy (IGRT) can assist in setting up the patient on each fraction and maintaining their position during treatment delivery.

A head and neck treatment course can last up to 7 weeks and patients commonly experience anatomical changes over the course of their treatment. For example, weight loss, changes in tumour or nodal volumes, shifting of fluids and muscle mass, and post-operative changes have been widely reported (see for example [4] and references therein). The potential clinical impact of anatomical changes on the dose distribution for both the PTV and the OARs has been explored in the literature (see for example Brouwer et al [5]). It is possible that the PTV coverage could be compromised [6], the maximum dose to the spinal cord could exceed tolerance, potentially resulting in paralysis, pain, and sensory deficits [7], and that sparing of other OARs could be ineffective, for example causing damage to the parotid glands leading to xerostomia [8]. Modern conformal techniques continue to improve dosimetry and minimise negative clinical sequelae but there remains scope for improvement.

Adaptive radiotherapy (ART) is an attractive solution; the patient’s plan can be adjusted to their changing anatomy, rather than assuming that it is identical to the pCT. ART can be either on-line, where any required changes to the plan are made on the same day, or off-line, where plan adaptations are performed after treatment and implemented for subsequent fractions. On-line ART is technically challenging, requiring accelerated re-planning, including contouring, re-optimisation of fields, and checking, all with the patient in the treatment position in the treatment room [9]. A range of off-line ART methods is reported in the literature for H&N patients. These include scheduling regular re-scan CTs (rCT) acquired on the CT-Sim in the treatment position, effectively acquiring new pCT scans [10–20], or using in-treatment-room imaging, for example verification MV-CTs [21–24], kV-CBCTs [19, 22, 25–30], and the relatively uncommon use of CT-on-rails [4, 31, 32]. The use of ART for H&N patients with MR-linacs has also recently been reported [33].

In practice, implementing ART can be time and resource intensive. Carrying out regular, potentially unnecessary, rCTs results in an additional patient appointment and additional patient dose, as well as extra resource and staff time. Using in-room verification imaging for ART exploits an existing step in patient treatment given that most centres now use daily or weekly CBCT for patient positioning, requiring no additional patient dose or appointments. However, the difficulty of calculating dose on CBCTs has been well-documented [34]. Proposed solutions include density overrides of the CBCT Hounsfield Units (HUs) [35], using CBCT anatomy-specific HU- Relative Electron Density (RED) calibration curves [36, 37], and exploiting deformable image registration (DIR) of the pCT to CBCT [25–27].

It has been reported that only a subset of the head and neck patient populations will benefit from ART [5, 21, 38], and identifying these patients prior to their radiotherapy treatment is difficult [22]. There is, however, the potential to better allocate the use of resource and staff time by selecting patients who will benefit from ART during treatment based upon a clinical trigger, for example a change in patient size that would impact upon the coverage of the PTV, spinal cord doses, or OAR doses.

Following our protocol, head and neck patients have an rCT when potentially unacceptable variations in anatomy from the pCT are observed on the kV CBCT used for verification imaging. This may include significant weight loss or changes in flexion or extension of the cervical spine that cannot be adjusted with set up. The patient’s original treatment plan is copied to the rCT scan using a rigid registration, using a bony match with further manual adjustments if required, to the pCT, and the dose to the spinal cord is evaluated. If the spinal cord dose is outside tolerance, then a re-plan will be carried out on the rCT. On some occasions, an rCT and re-plan will be required for other reasons, such as tumour growth requiring a new immobilisation device; in these cases, the clinical decision to re-plan has already been taken prior to the rCT being acquired. This work seeks to identify a clinical re-plan trigger based on the dose to the spinal cord, resulting in an improvement to the patient experience by reducing the numbers of unnecessary rCTs based on spinal cord dosimetry queries and the associated additional workload for radiographers, clinicians, physicists and dosimetrists.

## Methods

Thirty-five patients treated for cancer of the head and neck that required an rCT during the course of their treatment were identified for inclusion in this retrospective study. The patients were immobilised for the acquisition of their pCT, consisting of a helical scan with 2.5 mm slice width, and treatment using a thermoplastic mask. The patients were treated with VMAT (optimisation algorithm PRO 10028 (Varian Medical Systems, Palo Alto)) with one of the following dose prescriptions: 70.0 Gy in 35 fractions, 66.0 Gy in 33 fractions, or 60.0 Gy 30 fractions. Four patients were excluded from the study as their rCT was carried out prior to their first treatment fraction. The images analysed for each of the remaining 31 patients consisted of the pCT, the CBCT just prior to the acquisition of the rCT, and the rCT.

The volume of the body contour for each patient was evaluated on the pCT, CBCT, and rCT, to investigate potential correlation between the volume of the body contour, as a surrogate for anatomical changes, and spinal cord dose changes. ROIs were carefully created on each scan and the contours limited to within the ROIs, using the Contouring workspace of Varian Eclipse, so that the same scan length was compared.

Rigid registrations between the pCT and rCT, and pCT and CBCT, were created using the Image Registration module of Eclipse, or reviewed if already present, to enable copying of plans and contours. Using the registration, the original treatment plan with the full treatment prescription was copied to the CBCT and rCT. The spinal cord contours were created on the CBCTs by a single observer, by copying the original pCT spinal cord contour and adjusting the contour on each slice to account for any changes in spinal cord position. The spinal cord contours on the CBCT were verified by a clinician for a selection of the patients in the study. The dose for the full dose prescription of the treatment plans (to facilitate comparison between the cohort of patients) was re-calculated on the new images (CBCT and rCT) using Varian Eclipse AAA v13.6.23 (Varian Medical Systems). For the calculations on the CBCT, the standard CT HU-RED conversion curve was applied with no further density overrides. The D0.1cc to spinal cord was evaluated using the DVH for the doses from the CBCT and rCT.

The pCT was then deformed to the CBCT, creating a DIR, using the SmartAdapt® workspace of Varian Eclipse to form the deformed CT (dCT). The default deformable registration algorithm in SmartAdapt® is a modified demons algorithm [39]. The ROI for the DIR was carefully selected to obtain a satisfactory registration that covered the full extent of the high dose PTV, where possible. The DIRs were evaluated in line with the recommendations of TG132 [40]. The deformation vector field (DVF) created from the DIR was checked for non-physical results such as folding by viewing the deformation grid, which is deformed according to the DVF, and the deformation distance colour map showing the length of the deformation vectors. The DVF also warps the pCT contours; these were visually inspected for inconsistencies but not found to require adjustments. The dose was calculated on the dCT and the maximum dose to 0.1 cc of the spinal cord (D0.1cc) evaluated from the DVH.

The pCT spinal cord D0.1cc was compared to that calculated on the rCT, to evaluate whether these patients had an increase in spinal cord doses. The spinal cord D0.1cc calculated on the CBCT and dCT was compared to that calculated on the rCT, considered the gold standard, in order to identify the possibility of using these methods as a re-plan trigger. The spinal cord tolerance used for planning is D0.1 cc < 45.0 Gy.

Microsoft Excel and SPSS Statistics (IBM) were used to perform the statistical analysis, with paired sample tests used to examine the statistical significance of changes in the body contour and spinal cord. Where the distribution of the data was found to be normal on examination of histograms, q-q plots, and the results of a Kolmogorov-Smirnoff test, the student’s paired sample t-test was used. Where normality could not be assumed, a Wilcoxin signed rank test was carried out. Statistical significance was assumed with *p* < 0.05.

## Results

### Volume metrics

**Fig. 1:**
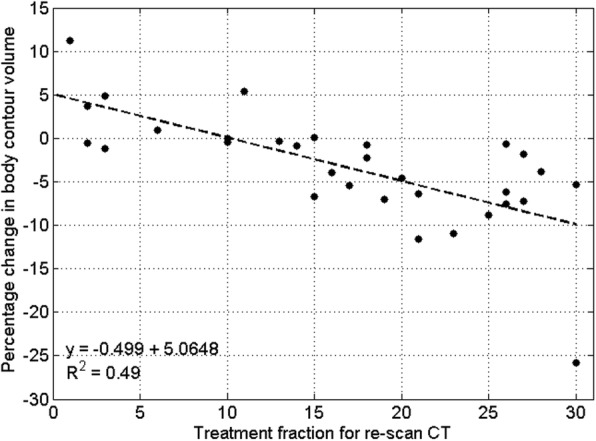
The percentage change in body contour volume between the pCT and rCT with the fraction of treatment for re-scan. There is a larger reduction in body contour volume as treatment progresses

All patients experienced a statistically significant change in the volume of the body contour between the pCT and rCT. For the majority of patients (25/31), the body contour decreased in volume (average 5.2 ± 5.5%, paired sample t-test p < 0.05) suggesting that the patients lost weight in this time. There is a weak correlation (R^2^ = 0.49) between fraction of re-scan and change in contour volume, with patients re-scanned later in their course of treatment exhibiting a larger reduction in contour volume (Fig. 1). Of the remaining six patients, one had almost no change in contour volume. The other five patients had an increase in body contour volume (average 4.4 ± 4.0%, paired sample t-test *p* < 0.05); for three patients this increase appears to be due to an increase in tumour volume. No correlation was found in this group of patients between change in volume of the body contour and change in spinal cord dose.

### Spinal cord doses: pCT to rCT

**Fig. 2:**
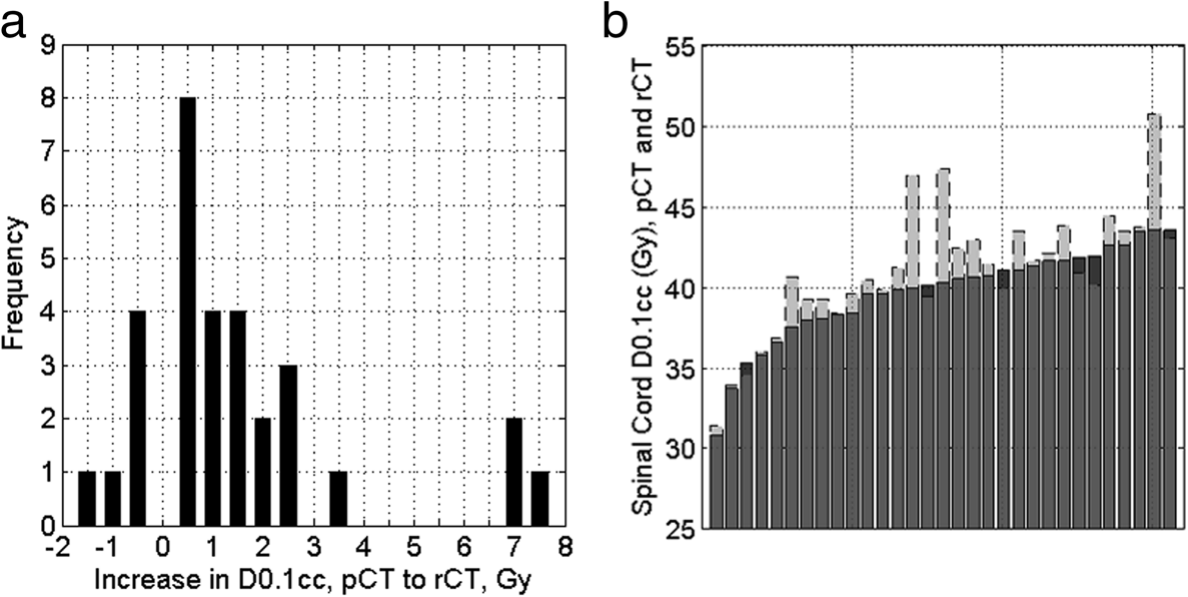
Spinal cord dose increases for the patients in this study. **a** shows the frequency distribution of the differences in spinal cord D0.1cc doses between pCT and rCT. The majority of patients experience a small change in spinal cord dose. The distribution is skewed by a small number of patients showing a large increase in D0.1cc. **b** shows the changing doses for each patient between the pCT and the rCT. The pCT dose is in grey with a solid outline and the rCT dose shown as a stacked light grey bar with a dashed outline. For the patients whose D0.1cc to spinal cord was reduced at the rCT with respect to the pCT, the decrease is shown as a dark outline over the initial pCT dose

Currently, patients in our centre will receive an ART re-plan according to the spinal cord dose on the rCT. The majority of the patients in this study experienced an increase in D0.1cc to the spinal cord from pCT to rCT, with the mean treatment fraction for the rCT being fraction 17 (range 1–30, standard deviation 9). The median spinal cord D0.1cc on the pCT was 40.3 Gy (interquartile range 3.6 Gy). The median dose increase to D0.1cc was 0.7 Gy (inter-quartile range 0.2–1.9 Gy, *p* < 0.05 Wilcoxin signed rank test). This data is shown in Fig. 2a as a frequency histogram. The majority of the patients have a relatively small change in spinal cord D0.1cc of ±2.5 Gy. The distribution is skewed by three patients who have spinal cord D0.1cc increases of ≥7.0 Gy; the same three patients exceeded the tolerance to D0.1cc of spinal cord of 45.0 Gy. Figure 2b shows the data presented as increases for each patient from the pCT dose.

### Spinal cord doses: calculating on the CBCT

**Fig. 3:**
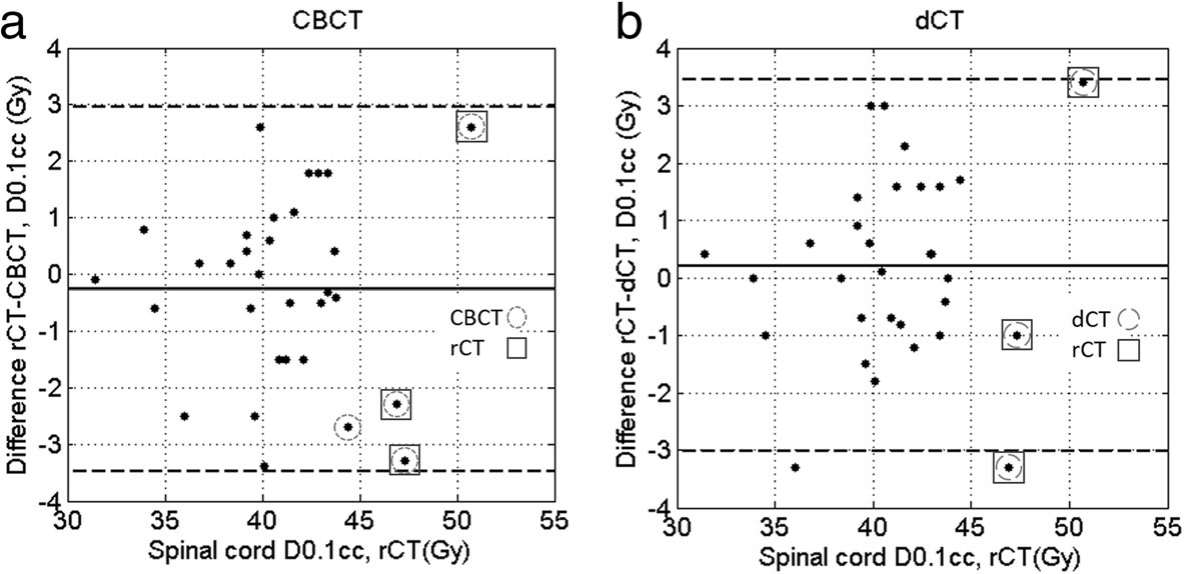
Bland-Altmann charts illustrating the difference between the D0.1cc to spinal cord for each patient as calculated on (a) the CBCT and (b) the dCT, compared to the gold standard of calculating on the rCT. The solid line indicates the mean difference and the dashed lines the 95% confidence intervals. On each panel, the data points representing patients triggered for a re-plan using the CBCT and dCT have been highlighted with a circle and compared to those triggered on the rCT, which are highlighted with a square

Figure 3a shows the results of calculating on the CBCT to estimate spinal cord D0.1cc, with the difference between calculating on the CBCT and the rCT plotted against the dose calculated on the rCT as the gold standard. All values calculated using the CBCT values agree with the rCT to within ±3.4 Gy.

### Spinal cord doses: calculating on the dCT

Figure 3b shows the results of calculating on the dCT to estimate spinal cord D0.1cc. The mean difference from the rCT is 0.2 Gy. All values calculated using the dCT agree with the rCT within ±3.4 Gy.

It appears from Fig. 3a and b that there could be a relationship between the magnitude of the change in spinal cord dose and the spinal cord dose on the rCT. Upon analysis, no correlation (R^2^ < 0.2) was found between the absolute change in spinal cord dose and the rCT spinal cord dose.

Although there is an uncertainty of ±3.4 Gy in determining the D0.1cc using the dCT or CBCT, both methods correctly identified three patients whose D0.1cc to the spinal cord exceeded the 45.0 Gy spinal cord tolerance. These same three patients experienced large increases in spinal cord D0.1cc, as seen in Fig. 2.

Adopting a trigger level of D0.1cc = 45.0 Gy to the spinal cord based on the dCT calculations resulted in no false positives or negatives, and one false positive using the CBCT calculations. Considering the uncertainty in the dose calculation, a conservative action level on the dCT/CBCT of D0.1cc = 42.0 Gy could be adopted. With this criteria, 20/28 patients would have been spared an rCT using the dCT, and 17/28 patients when using the CBCT.

## Discussion

For a group of 31 head and neck patients, patient size, as represented by the volume of the patient’s body contour, cannot be considered as a useful trigger for ART based on spinal cord doses. However, calculating on the patient’s verification CBCT images, or on the pCT deformed to the CBCT, can be used to provide an estimate of spinal cord doses and thus trigger an adaptive re-plan where necessary. This will reduce the number of unnecessary additional CT scans, especially considering that the number of patients requiring an ART plan due to spinal cord doses is small.

It has been proposed that weight loss in head and neck patients influences patient shape (e.g. [4, 16]) and positioning [11, 41], and therefore has the possibility of affecting the dose distribution with potential increases to OARs (for example [5]). In particular, weight loss has been correlated to an increase in the maximum dose to the spinal cord [42] and doses to the parotid glands [23, 42]. A number of authors implement ART in H&N patients according to a change in body contours; for example a 5 mm margin around the target volume [25] or 1 cm change anywhere in the body contour [22, 43].

Whilst we observed statistically significant changes in the body contour over time, suggesting weight loss in line with other published data [21, 44], there was no significant correlation found between change in contour volume and change in dose to the spinal cord. This is in agreement with results published by Noble et al, who found no correlation between shape change and change in spinal cord doses [21]. This suggests that change in patient shape cannot be used as a clinically useful re-plan trigger according to spinal cord dose. Similarly, in this group of patients some re-plans were carried out for other reasons, including disease progression and requirement of a new immobilisation mould. A larger change in body contour volume was not found to correlate with re-plan requirement for any reason. This finding is in agreement with Hansen et al [44], who observed no statistically significant correlations in any of their studied metrics between patients re-planned due to weight loss and those re-planned for other reasons.

In this group of patients, there was a small but statistically significant change in D0.1cc to the spinal cord of 0.7 ± 2.2 Gy, in agreement with values in the literature where average increases in D_max_ were reported in the range 1.1–1.4 Gy [14, 21, 28, 45]. 81% of the patients had an increase in dose to cord; this is comparable to values reported in the literature, for example Wang et al [42] reported 87% and Hansen et al. [44] 100% of patients showing an increase in dose to the cord.

For three patients there was an increase in spinal cord dose between the pCT and rCT of 7.0–7.2 Gy, resulting in doses exceeding the cord tolerance for the plan. Only one of these three patients had an initial spinal cord dose close to the planning tolerance, at 43.5 Gy. This has also been reported in the literature, with between 5 and 30% of patient cohorts exhibiting increases that push the spinal cord dose out of tolerance [30, 46–48]. On review of the CT and CBCT imaging for these three patients, the large dose increases can be attributed to significant positioning changes due to curvature of the spine, pushing the spinal cord into a high dose region.

Using the CBCT and dCT to calculate D0.1cc to spinal cord and trigger a re-plan is feasible for these patients. The three patients with large spinal cord dose increases, resulting in tolerance being exceeded, were correctly identified using both methods. Variations in calculated doses on the CBCT with respect to CT are ±3.4 Gy, in line with Fotina et al [35], who reported a 5% variation in target coverage on the CBCT, and Naufal et al [49] who found a 7% variation in H&N CBCT calculations.

The method presented here involves recalculating the entire dose prescription on the rCT. Calculating instead a cumulative spinal cord D0.1cc from the pCT and rCT results in one patient exceeding the D0.1cc tolerance of 45.0 Gy with an increase of 6.8 Gy. Despite not exceeding the planning tolerance, the remaining two of the three patients previously identified also exhibited large increases in spinal cord doses of 3.4 and 3.6 Gy, well above the median cumulative increase of 0.4 Gy. A cumulative analysis assumes that the patient’s spinal cord dose will remain stable for the remainder of their treatment. Calculating the entire prescription therefore presents a conservative method by which to trigger a re-plan for the most at-risk patients, regardless of the time point in the patient’s treatment course.

In this study, there were only three patients with spinal cord doses above the tolerance of D0.1 cc of 45 Gy against which to test our re-plan trigger. This data, however, was gathered over a period of 2.5 years with on average 17 H&N VMAT plans created per month; this supports the conclusion that selectivity for ART in this cohort of patients is of utmost importance to achieve the best balance of additional workload to clinical benefit. This will result in a potential reduction from 9 to 1% of H&N patients requiring a rCT, reducing the need for additional dose to these patients. Furthermore, we have only examined the dose to the spinal cord. This approach was taken as our clinical decision-making is based upon spinal cord doses on the rCT. Other publications have reported in depth the positional and dosimetric changes to other OARs, with the parotid glands the OAR of focus. Dosimetric sparing of the parotids has been reported to be approximately 1 Gy to mean parotid dose [12, 19, 32], although one study reported increases to the parotid mean dose of, on average, 4 Gy to 60% of the patient cohort [50]. The clinical benefit of such sparing with re-planning has been questioned [19], with the NTCP increase for grade 3 xerostomia estimated at 0.03% in one study [12]. There is again evidence that these dosimetric increases push the OAR dose above tolerance in only a small minority of cases [47]. Since the conclusion of this study, our centre has moved to daily CBCT imaging for H&N patients. With more frequent imaging and therefore an improving evidence base, other lower priority OARs that may benefit from ART, for example the parotid glands, could also be evaluated using our method.

## Conclusions

For a group of 31 H&N patients, change in body contour volume was not found to indicate the necessity of a re-plan according to spinal cord dose tolerances. However, significant positional changes such as neck flexion and extension can. The spinal cord doses as calculated on the patients’ verification CBCT image or the pCT deformed to the CBCT anatomy can be used as a trigger to identify the subset of patients that require an adaptive re-plan, reducing the need to acquire unnecessary additional CT scans. The number of patients that require a re-plan due to spinal cord doses is small, indicating that ART for spinal cord doses is necessary only for a minority of head and neck patients, despite the large anatomical changes that they experience.

## Data Availability

The dataset supporting the conclusions of this article is included within the article. Additional data are available from the corresponding author on reasonable request.

## Abbreviations

ART: Adaptive radiotherapy
CBCT: Cone beam CT
dCT: Deformed CT
DIR: Deformable image registration
DVF: Deformation vector field
DVH: Dose-volume histogram
H&N: Head and neck
HU: Hounsfield units
OAR: Organ at risk
pCT: Planning CT
PTV: Planning target volume
rCT: Re-scan CT
RED: Relative electron density
